# A New Role for a Protein Involved in Energy Metabolism

**DOI:** 10.1371/journal.pbio.0030133

**Published:** 2005-03-15

Adjusting to life after birth takes a lot of energy. One way cells meet increased demand is by ramping up synthesis of mitochondria, the cells' power generators This ability to increase mitochondria becomes limited in a variety of diseases including diabetes and heart failure. Therefore, it is important to identify the factors that control mitochondrial function. One way researchers have searched for candidate proteins that play a role in this process is by overexpressing proteins in targeted cells to see what happens. That's how several previous studies concluded that a protein called PGC-1α triggers pathways that promote mitochondrial synthesis and regulate both mitochondrial activity and energy metabolism.

In a new study, Daniel Kelly and colleagues took a different approach. Rather than increasing the protein's activity, they blocked it. To do that, Kelly and colleagues engineered “knockout” mice that lack functional copies of the *PGC-1*α gene. PGC-1α, they found, isn't absolutely required for mitochondrial biogenesis but plays a vital role later in life by “boosting” the ability of cells to increase mitochondrial function in response to the shifting energy demands and physiological stresses encountered after birth.

Though leaner than the control mice soon after birth, by 18 weeks the female knockouts were slightly heavier and had more body fat, even though their food intake and activity levels matched the controls. Knockout mice had observable growth defects in skeletal and heart muscle—tissues with high mitochondrial energy requirements—were less active and more easily fatigued than the controls, and had abnormal heart rates after physical exertion. And their livers showed a propensity to accumulate fat because of abnormal mitochondria.[Fig pbio-0030133-g001]

**Figure pbio-0030133-g001:**
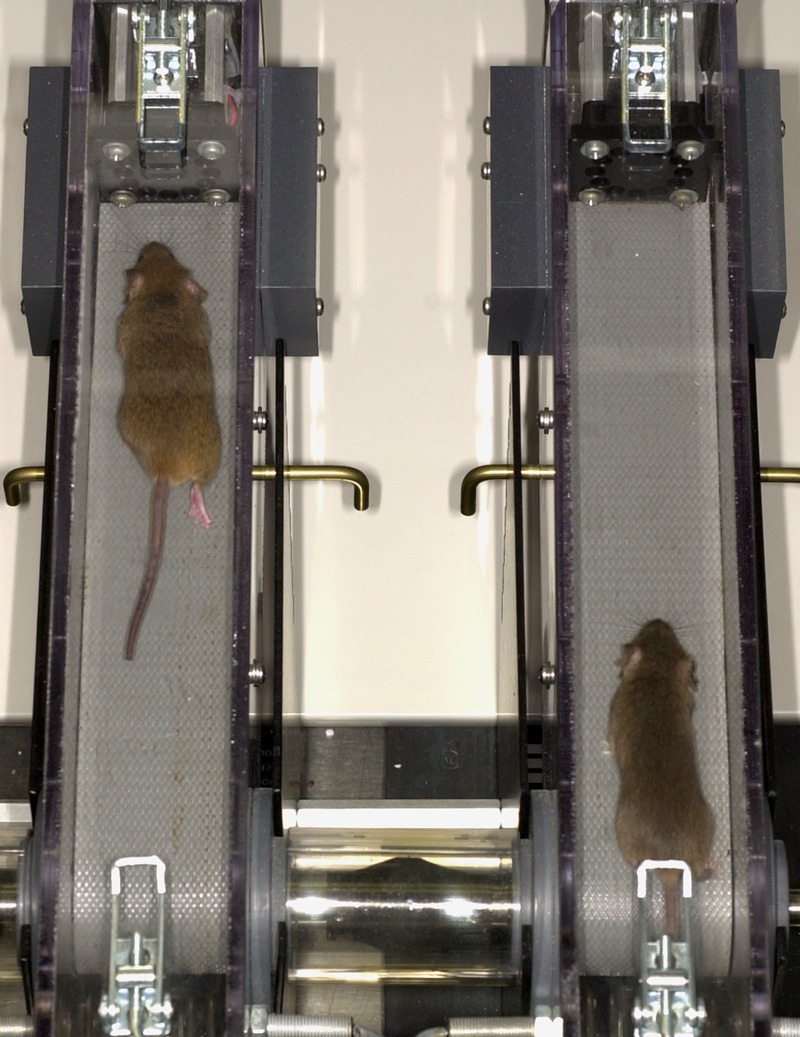
PGC-1α deficient mice can't keep pace (Photo: MedPic, Washington University)

Altogether, these results demonstrate PGC-1α's critical role in regulating the adaptive metabolic responses required by the increasing energy demands and changing physiological stimuli associated with a growing organism. The increased fat stores and weight gain in the knockout mice, the authors propose, could result from a systemic reduction in energy use, related to defective mitochondria. Given the recently reported link between *PGC-1α* mutations and human obesity and diabetes, this connection will likely trigger further investigations. And given the pivotal role mitochondria play in a wide range of organs, this mouse model could help shed light on metabolic defects associated with a wide range of diseases.

Interestingly, another group, led by Bruce Spiegelman, reported on a *PGC-1α* knockout model last year. Their mice share traits with the mice described here, but also exhibit a number of contrasting traits, including hyperactive, lean males, which the Spiegelman group attributed to a neurological defect. Kelly and colleagues speculate on possible causes for the differences in the results of the two studies, but only direct comparison of both mouse models will explain the inconsistencies.

